# miR-155 Regulates IL-10-Producing CD24^hi^CD27^+^ B Cells and Impairs Their Function in Patients with Crohn’s Disease

**DOI:** 10.3389/fimmu.2017.00914

**Published:** 2017-08-03

**Authors:** Yingxia Zheng, Wensong Ge, Yanhui Ma, Guohua Xie, Weiwei Wang, Li Han, Bingxian Bian, Li Li, Lisong Shen

**Affiliations:** ^1^Department of Laboratory Medicine, Xin Hua Hospital, Shanghai Jiao Tong University School of Medicine, Shanghai, China; ^2^Institute of Biliary Tract Diseases Research, Shanghai Jiao Tong University School of Medicine, Shanghai, China; ^3^Department of Gastroenterology, Xin Hua Hospital, Shanghai Jiao Tong University School of Medicine, Shanghai, China

**Keywords:** miR-155, CD24^hi^CD27^+^ B cells, B10 cells, Crohn’s disease, IL-10

## Abstract

Regulatory interleukin-10 (IL-10)-producing B cells (B10 cells) play a critical role in preventing and curing autoimmune diseases in experimental mouse models. However, the precise cellular and molecular mechanisms of action of B10 cells in humans, especially in patients with Crohn’s disease (CD), remain to be determined. miR-155 regulates many physiological and pathological conditions, including inflammation such as that in CD. In this study, we aimed to explore the effect of miRNA-155 on IL-10 production by B cells in healthy controls (HCs) and CD patients. Interestingly, we found that CD24^hi^CD27^+^ B cells express high levels of miRNA-155 and IL-10, which are positively correlated. Additionally, CD24^hi^CD27^+^ B cells express higher levels of Toll-like receptor 9 than those found in other B cell subsets. Overexpression of miRNA-155 promotes IL-10 production, while inhibition of miRNA-155 decreases IL-10 production. We determined that miR-155 directly inhibits the expression of Jarid2, which reduces H3K27me3 binding to the *IL10* promoter and increases *IL-10* gene expression. In coculture systems, the CD24^hi^CD27^+^ B cells from HCs suppressed the secretion of TNFα and IFNγ by monocytes and T cells, respectively. However, the number and function of CD24^hi^CD27^+^ B cells from CD patients were decreased. Moreover, we found that miR-155 induces CD24^hi^CD27^+^ B cells to produce higher levels of TNFα instead of IL-10 in CD patients than in the controls and that the increased number of IL-10^+^TNFα^+^ B cells reduces the induction of Foxp3 expression and the inhibition of IFNγ production by CD4^+^CD25^−^ T cells, as well as TNFα production by monocytes. Our study demonstrates the critical role of miRNA-155 in the regulation of IL-10 production by B cells and reveals the novel molecular mechanism underlying the functional impairment of B10 cells in CD patients. Our study has the potential to drive the development of B10 cell-based strategies to ameliorate disease progression in CD patients.

## Introduction

Many studies have conclusively demonstrated the significance of interleukin-10 (IL-10)-producing B cells (B10 cells) in diverse murine models and in human research on autoimmunity, infection, and cancer (1–4). The main mechanism of suppression by B10 cells is mediated by the release of IL-10, leading to the inhibition of IFNγ, TNFα, and IL-17 production by immune cells (5–7). In addition, it has been reported in mice that B10 cells are enriched in B cells that express CD1d and CD5, while in humans, B10 cells are enriched in B cells that express CD24, CD27, and CD38 (8–10). Toll-like receptor (TLR) agonists, such as CpG oligonucleotides, potently induce B cells to produce IL-10 by activating STAT3 phosphorylation in human B cells (11). A proliferation-inducing ligands also promote the production and regulatory functions of IL-10 in human B cells (12). Additionally, IL-35 induces regulatory B cells to suppress autoimmune diseases (13). However, the signaling pathways responsible for the development and function of B10 cells in humans are largely unknown; in particular, the roles of microRNAs (miRNAs) and chromatin regulators in these processes have not yet been demonstrated.

Crohn’s disease (CD), characterized by inappropriate and exacerbated immune responses within the gastrointestinal tract, is the most common form of inflammatory bowel disease (IBD). The pathogenesis of CD has been associated with complex interactions among the host’s genetic susceptibility, environmental factors, and immune cell imbalance, but most of the molecular mechanisms remain unknown (14–16). Recent studies have found that mutations in genes encoding IL-10 and IL-10R are associated with a very early-onset form of IBD (17). Regulatory B cells producing IL-10 have been shown to play an important role in maintaining intestinal homeostasis, and a lack of B10 cell-mediated suppression has been associated with CD development (18). Sattler et al. reported that B10 cells effectively attenuate mucosal inflammatory responses in the gut (19), and CD patients are characterized by a decrease in regulatory B cells (20, 21). However, the mechanisms underlying the reduction in the number and function of B10 cells in CD patients have not been fully investigated.

MicroRNAs can post-transcriptionally modulate the expression of multiple target genes and play a critical role in the development of inflammatory diseases (22, 23). It has been reported that miR-155 is a typical multifunctional miRNA involved in hematopoiesis, inflammation and immune responses and whose expression is induced by inflammatory cytokines and TLR ligands (24, 25). Recently, the impact of miRNA-155 on the colonic mucosa of patients with ulcerative colitis (UC) was studied, and it was revealed that miR-155 modulates the inflammatory phenotype of intestinal myofibroblasts by targeting SOCS1; miR-155 has also been shown to be involved in the pathogenesis of UC by targeting FOXO3a (26, 27). Thelma et al. reported that miR-155 activates cytokine expression in Th17 cells (28). However, until now, no study has reported the relationship between miR-155 and B10 cells, especially in patients with CD. Thus, the role of miR-155 in modulating the development and function of B10 cells warrants further study.

Therefore, the purpose of the current study was to count and characterize B10 cells in healthy subjects and CD patients. Additionally, we wanted to explore the effect of miRNA-155 in modulating the development and function of B10 cells. Our study reveals the critical role of miR-155 in the regulation of IL-10 production in B cells and demonstrates novel molecular mechanisms underlying the impaired number and function of B10 cells in CD patients, which will promote the development of B10 cell-based strategies to prevent disease progression in CD patients.

## Materials and Methods

### Patients

We examined B10 cells in the peripheral blood of patients with active CD (*N* = 30; 16 men; 14 women; mean age: 32.5 ± 3.0 years; range: 14–52 years). Samples from healthy individuals (*N* = 50; 25 men; 25 women; mean age: 32.1 ± 2.5 years; range: 18–56 years) served as controls. Active CD was prospectively defined as a Crohn’s disease activity index (CDAI) >150. All the patients provided written informed consent to participate in the study, which was carried out in accordance with the principles of the Declaration of Helsinki and was approved by the ethics committee of Xinhua Hospital.

### Flow Cytometric Analysis

For intracellular cytokine staining, cells were stimulated with a cell stimulation cocktail plus protein transport inhibitors (eBioscience) for 5 h. Then, the cells were fixed and permeabilized with Cytofix/Cytoperm buffer, and intracellular cytokines were stained with antibodies against IL-10, IFNγ, and TNFα (eBioscience). Flow cytometric analysis was performed with a FACS Canto II instrument (BD Bioscience) and FlowJo software (TreeStar).

### Cytokine Production Measurements

Interleukin-10 and TNFα levels were measured with ELISA kits according to the manufacturer’s instructions (R&D Systems).

### Immunoblot Analysis

Cells were directly lysed and subjected to 10% SDS-PAGE. Immunoblotting was performed after transferring the proteins onto nitrocellulose membranes (Schleicher & Schuell Microscience) with a Mini Trans-Blot apparatus (Bio-Rad). After 2 h of blocking, the membranes were incubated overnight at 4°C with the following specific primary antibodies: anti-Jarid2 (Abcam), anti-p-STAT3, anti-STAT3 (Cell Signaling), and anti-β-actin Abs (Sigma-Aldrich). After the membranes were washed, subsequent incubations with the appropriate HRP-conjugated secondary antibodies were conducted for 1 h at room temperature; after extensive washing, the signals were visualized with an ECL substrate (Pierce Chemical).

### Real-time PCR

Expression levels of miR-155, IL-10, TNFα, and Jarid2 were measured by quantitative RT-PCR (qRT-PCR) using a SYBR green-based real-time quantitative PCR assay (Qiagen). The data were collected and quantitatively analyzed using an ABI Prism 7900 Sequence Detection System (Applied Biosystems). The relative level of miR-155 was normalized to that of small nuclear RNA U6, which is a ubiquitously expressed small nuclear RNA that has been widely used as an internal control. Primers for miR-155 and the small nuclear RNA U6 were obtained from Invitrogen. The sequences of the primers are as follows: miR-155, forward 5′-TTA ATG CTA ATC GTG ATA GGG G-3′ and reverse 5′-CGA ATT CTA GAG CTC GAG GCA GG-3′; U6, forward 5′-CTC GCT TCG GCA CA-3′ and reverse 5′-CGA ATT CTA GAG CTC GAG GCA GG-3′; IL-10, forward 5′-CTT CGA GAT CTC CGA GAT GCC TTC-3′ and reverse 5′-ATT CTT CAC CTG CTC CAC GGC CTT-3′; Jarid2, forward 5′-GCT TCC CAC CAG GAT GAC AG-3′ and reverse 5′-CCA AGG AGC CCA TTC ACA GT-3′; TNFα, forward 5′-CAC CAC TTC GAA ACC TGG GA-3′ and reverse 5′-AGG AAG GCC TAA GGT CCA CT-3′; and GAPDH, forward 5′-GCC ACC CAG AAG ACT GTG GAT GGC-3′ and reverse 5′-CAT GTA GGC CAT GAG GTC CA C CAC-3′. The data are presented according to the following equations: target miRNA expression = 2ΔCt, with ΔCt = (U6 Ct-target miRNA Ct), or target mRNA expression = 2ΔCt, with ΔCt = (GAPDH Ct-target mRNA Ct).

### CFSE Labeling

We added CFSE (Sigma-Aldrich) to B cell suspensions at a final concentration of 2 µM for 8 min at 37°C; then, we washed the cells three times with PBS and resuspended them in complete RPMI medium.

### *In Vitro* Coculture Assay

Cocultures of 2.0 × 10^5^ B cells and 2.0 × 10^5^ CD4^+^ CD25^−^ T cells stimulated for 72 h with plate-bound CD3 mAb (0.5 µg/mL) were activated with stimulation cocktail plus protein transport inhibitors (eBioscience) for the last 5 h. In addition, 2.0 × 10^5^ B cells and 2 × 10^5^ CD14^+^ monocytes cocultured for 24 h were stimulated with LPS (100 µg/mL) for the final 5 h. The cells were stained for surface markers, permeabilized, stained intracellularly for IFNγ or TNFα and Foxp3 and analyzed by flow cytometry.

### miRNA Mimic or Inhibitor Transfection

Isolated B cells (3 × 10^6^ B cells, Miltenyi Biotec) in 100 µL of Amaxa mix were electroporated with 300 nM miR-155 mimic or inhibitor or control (Sigma) according to the manufacturer’s instructions. Six hours after transfection, we added 100 nM CpG oligonucleotides to the culture. Then, we harvested the cells for further analysis.

### siRNA Knockdown

We performed RNA interference experiments using electroporation (Amaxa) according to the manufacturer’s protocol. Briefly, we mixed 300 nM Jarid2-specific siRNA or control siRNA (Sigma) with 3 × 10^6^ B cells in 100 µL of Amaxa mix and transfected the cells via electroporation according to the manufacturer’s instructions. Six hours after transfection, we added 100 nM CpG oligonucleotides to the culture. Then, we harvested the cells for further analysis.

### Isolation of Cytokine-Producing B Cells

We followed a previously described protocol to isolate cytokine-producing B cells (10, 29). First, B cells were pre-enriched via depletion of non-B cells (Miltenyi Biotec) and cultured for 2 days under stimulation with 100 nM CpG oligonucleotides. Second, stimulation cocktail (eBioscience) was added to the cultures for 3 h to induce IL-10 secretion. Third, the viable cytokine-producing B cells were specifically isolated using a cytokine secretion assay according to the manufacturer’s instructions. Briefly, the pre-enriched B cells were incubated with IL-10 and TNFα catch reagents. The cells were subsequently labeled with IL-10 detection antibody or TNFα detection antibody conjugated to PE or APC, respectively. The IL-10- and TNFα-secreting cells were then sorted by FACS (BD Aria II). The purity of the cells was further confirmed by measuring the expression of IL-10 and TNFα by q-RT-PCR.

### ChIP

ChIP assays were performed using a ChIP assay kit (Millipore) with modifications. Isolated B cells (5 × 10^6^ cells) were fixed in 1% formaldehyde, and the chromatin was sonicated and pre-cleared by incubation with Protein A/G agarose/salmon sperm DNA (Millipore). The precleared chromatin was immunoprecipitated with antibodies against H3K27me3 (Abcam) overnight at 4°C or mouse IgG monoclonal antibody followed by incubation with Protein A/G agarose/salmon sperm DNA for 1 h. The immunoprecipitates were denatured, and the DNA was purified. The amount of immunoprecipitated DNA was quantified by real-time PCR using SYBR Green and the ABI PRISM 7500 Sequence Detection System (Applied Biosystems). The primers used for the PCR analysis of the *IL-10* promoter locus are as follows: forward: 5′ to 3′, CCA GGT AGA GCA ACA CTC; reverse: 5′ to 3′ CAG GCT CCT TTA CCC CGA TT.

### Statistics

We used one-way analysis of variance to initially determine whether an overall statistically significant difference existed before using Tukey’s *post hoc* test and the two-tailed paired or unpaired Student’s *t*-test to analyze the differences between two groups. The results are presented as the means ± SEM. A value of *P* < 0.05 was considered statistically significant.

## Results

### miR-155 Expression Is Correlated with IL-10 Production in CD19^+^CD24^hi^CD27^+^ B Cells

**Figure 1:**
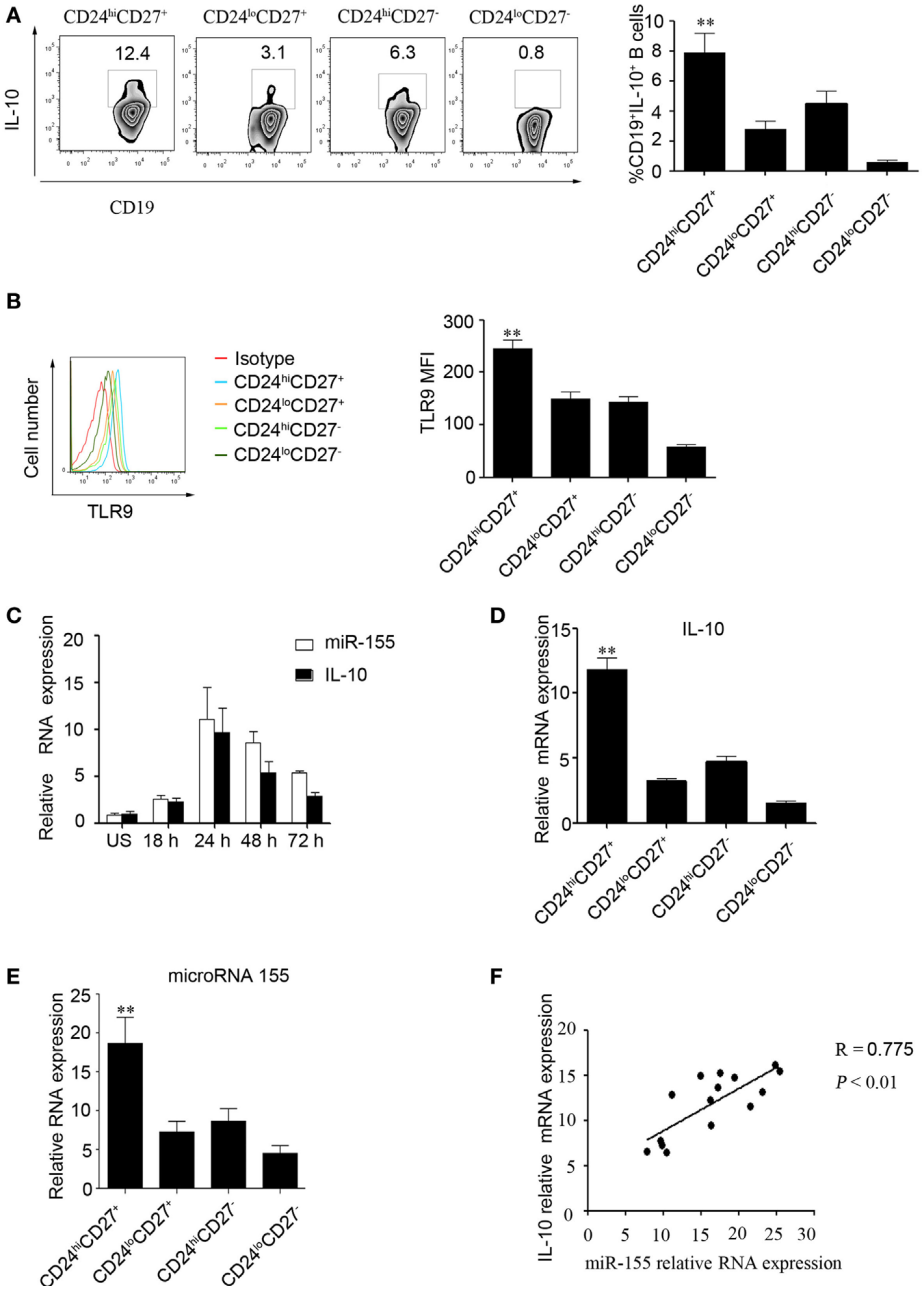
miR-155 expression is correlated with interleukin-10 (IL-10) production in CD19^+^CD24^hi^CD27^+^ B cells. **(A)** Healthy CD19^+^ B cells (*N* = 10) were cultured with 100 nM CpG oligonucleotides for 48 h with the stimulation or inhibition cocktail added during the final 5 h of culture, and the percentage of IL-10^+^ B cells within the indicated B cell subsets was determined (left panel) by flow cytometry. The data in the right panel are shown as the mean ± SEM. **(B)** Healthy CD19^+^ B cells (*N* = 10) were cultured with 100 nM CpG for 48 h, and the percentages of TLR9-expressing B cell subsets were determined by flow cytometry (left panel). The data in the right panel are shown as the mean ± SEM. **(C)** CD19^+^CD24^hi^CD27^+^ B cells were sorted and stimulated with or without 100 nM CpG oligonucleotides at different times, and the expression of miR-155 and IL-10 was analyzed by Q-PCR. **(D,E)** Different B cell subsets (*N* = 5) from healthy controls (HCs) were sorted and stimulated with 100 nM CpG oligonucleotides for 24 h; IL-10 **(D)** and miR-155 **(E)** levels were analyzed by Q-PCR. **(F)** Correlation between IL-10 and miR-155 gene levels in the CD19^+^CD24^hi^CD27^+^ B cells from HCs (*N* = 15). The *R* value represents the calculated regression coefficient. The *P*-value indicates the correlation index between the groups. **P < 0.01.

First, we used two markers, CD24 and CD27, to determine levels of IL-10 production in different B cell subsets (10). The gating strategies and isotype control are shown in Figure S1 in Supplementary Material. As reported by other studies, we found that the CD24^hi^CD27^+^ B cell subset produced higher levels of IL-10 after stimulation with CpG oligonucleotides for 48 h compared to those produced by other B cell subsets (Figure 1A). In addition, these data were supported by the TLR9 expression measured in different B cells subsets (Figure 1B); the data show that the CD24^hi^CD27^+^ B cells, which are more sensitive to stimulation by CpG oligonucleotides, expressed higher levels of TLR9 than other B cell subsets. Isolated human CD19^+^ B cells and were stimulated with CPG or not for 48 h, the miRNA profiles of the cells were examined, and we found that miR-155 was significantly increased after CPG stimulation (Figure S2 in Supplementary Material). Next, we isolated different B cell subsets according to the CD24 and CD27 markers and analyzed the expression of IL-10 and miR-155 using Q-PCR. We observed that the expression of miR-155 and IL-10 increased over time with the stimulation of CD24^hi^CD27^+^B cells by CpG oligonucleotides in culture; the expression reached its highest level at 24 h before gradually decreasing (Figure 1C). When cultured for 24 h, the CD24^hi^CD27^+^ B cells expressed higher levels of IL-10 and miR-155 than other B cell subsets (Figures 1D,E). In addition, IL-10 expression levels were significantly correlated with those of miR-155 in the CD24^hi^CD27^+^ B cell subset (*R* = 0.775, *P* < 0.01) (Figure 1F). These data suggest that the CD24^hi^CD27^+^ B cell subset expresses high levels of miR-155, which are correlated with IL-10 production.

### miR-155 Increases the Expression of the IL-10 Gene in B Cells by Regulating the DNA-Binding Protein Jarid2

**Figure 2:**
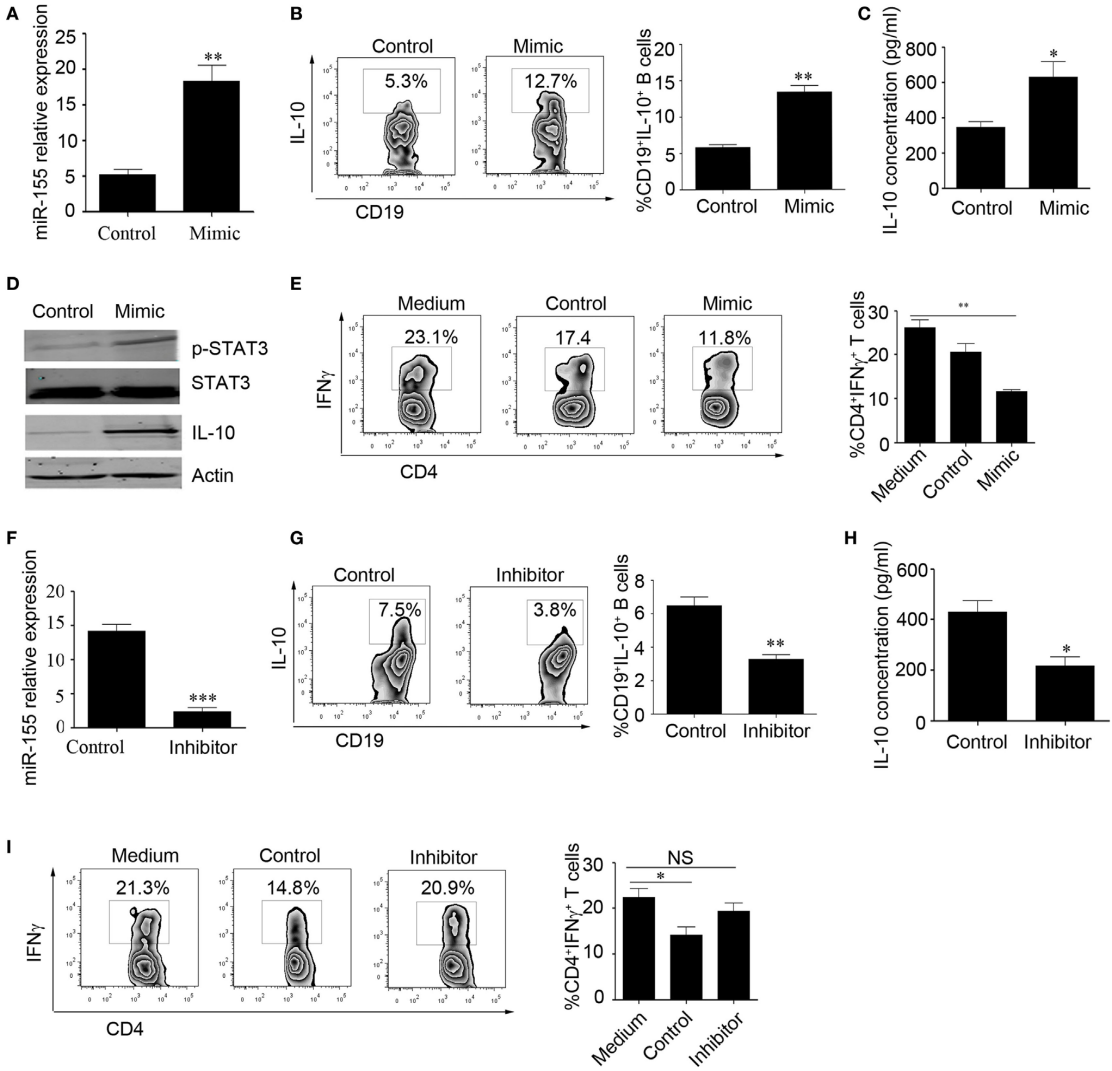
miR-155 activates interleukin-10 (IL-10) gene expression in B cells. **(A–E)** CD19^+^ B cells isolated from healthy controls (*N* = 3) were electroporated with miR-155 mimic or control and stimulated with 100 nM CpG oligonucleotides. **(A)** miR-155 levels were measured by Q-PCR after 24 h of culture. IL-10 expression was measured by flow cytometry **(B)** and IL-10 concentration was measured by ELISA **(C)** after culturing the cells for 48 h. **(D)** p-STAT3 and IL-10 expression were measured by Western blotting; STAT3 and β-actin were used as loading controls. **(E)** B cells were cocultured with CD3 mAb-stimulated CD4^+^CD25^−^ T cells isolated from healthy controls (HCs) for 48 h. IFNγ expression in the CD4 T cells was analyzed by FACS. **(F–I)** CD19^+^CD24^hi^CD27^+^ B cells isolated from healthy controls (*N* = 3) were electroporated with miR-155 inhibitor or control and stimulated with 100 nM CpG oligonucleotides. **(F)** miR-155 levels were measured by Q-PCR after 24 h of cell culture. **(G)** IL-10 expression was measured by flow cytometry, **(H)** and IL-10 concentration was measured by ELISA after culturing the cells for 48 h. **(I)** B cells were cocultured with CD3 mAb-stimulated CD4^+^CD25^−^ T cells isolated from HCs for 48 h. IFNγ expression in the CD4 T cells was analyzed by FACS. The data shown are the mean ± SEM. The asterisks represent statistical significance between the groups. *P < 0.05; **P < 0.01.

Next, we explored the effect of miR-155 on the regulation of IL-10 production. We isolated CD19^+^ B cells from healthy controls (HCs) and overexpressed miR-155 mimic or control using electroporation and stimulated the cells with CpG oligonucleotides for an additional 48 h. The survival of B cells was not significantly changed after transfection with the miR-155 mimic or control (Figure S2A in Supplementary Material). We found that the miR-155 expression level was significantly increased in the mimic-transfected group compared to that in the control group (18.33 ± 2.23 and 5.18 ± 0.77%, respectively), as well as the IL-10 production level (Figures 2A–C). As STAT3 phosphorylation is important for IL-10 production (11), we measured the phosphorylation of STAT3 in B cells overexpressing miR-155. By Western blot, we observed that after overexpression of miR-155 mimic in B cells, STAT3 phosphorylation and IL-10 expression were significantly increased (Figure 2D). We also conducted functional assays and found that when B cells had overexpression of miR-155 and were cocultured with purified CD4^+^T cells, INFγ production by T cells was significantly reduced (Figure 2E). In contrast, when we sorted CD24^hi^CD27^+^ B cells from the HCs and electroporated them with miR-155 inhibitor, the survival of B cells was not significantly changed after transfection with the miR-155 inhibitor or control (Figure S2B in Supplementary Material). We found that the expression level of miR-155 was decreased (14.11 ± 1.050 and 2.318 ± 0.6336%, respectively) and that the production level of IL-10 was significantly reduced compared to the corresponding levels in the control group (Figures 2F–H). Also, the inhibition of INFγ production by T cells was significantly decreased when CD24^hi^CD27^+^ B cells were transfected with miR-155 inhibitor (Figure 2I). These data demonstrate that miR-155 regulates IL-10 production in B cells, as well as B cell function.

**Figure 3:**
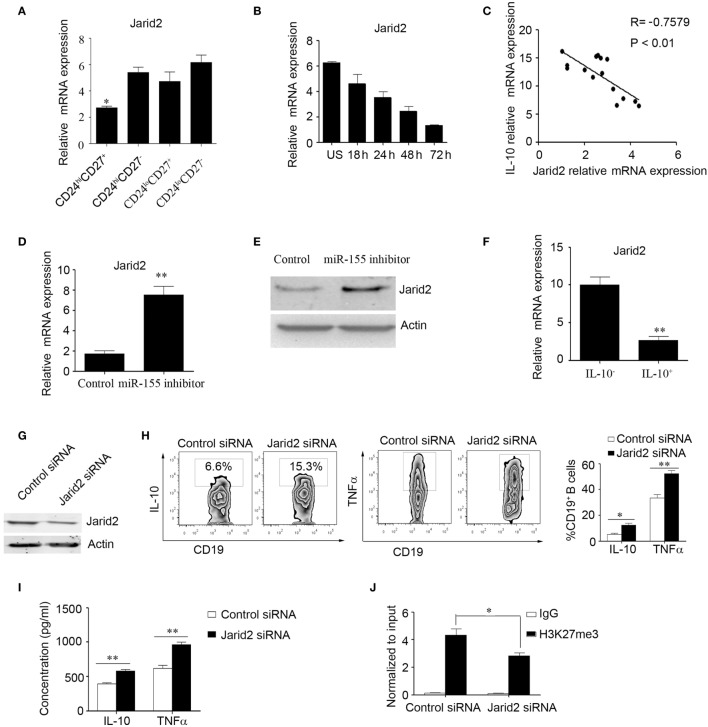
miR-155 increases interleukin-10 (IL-10) gene expression in B cells by regulating the DNA-binding protein Jarid2 and the repression of IL-10. **(A)** Different B cell subsets were sorted and stimulated with 100 nM CpG oligonucleotides for 24 h. Jarid2 gene expression was analyzed by Q-PCR. **(B)** CD19^+^CD24^hi^CD27^+^ B cells were sorted and stimulated with or without of 100 nM CpG oligonucleotides at different times. Jarid2 gene expression was analyzed by Q-PCR. **(C)** Correlation of IL-10 gene levels with Jarid2 gene levels in CD19^+^CD24^hi^CD27^+^ B cells (*N* = 15). The *R* value represents the calculated regression coefficient. The *P*-value indicates the correlation index between the groups. **(D,E)** CD19^+^CD24^hi^CD27^+^ B cells isolated from healthy controls (*N* = 4) were electroporated with miR-155 inhibitor or control and stimulated with 100 nM CpG oligonucleotides. **(D)** Jarid2 gene expression levels were measured by Q-PCR after 24 h of cell culture. **(E)** Jarid2 expression levels were measured by Western blotting; β-actin was used as the loading control. **(F)** CD19^+^IL-10^+^ B cells or CD19^+^IL-10^−^ B cells (*N* = 3) were isolated as described in the Materials and methods, and IL-10 gene expression was measured by Q-PCR. **(G–J)** CD19^+^ B cells isolated from healthy controls were electroporated with Jarid2-specific siRNA or control siRNA and stimulated with 100 nM CpG oligonucleotides. **(G)** Jarid2 expression was measured by Western blot after 48 h of cell culture; β-actin was used as the loading control. **(H)** IL-10 and TNFα expression in B cells was measured by FACS, **(I)** and the IL-10 and TNFα concentrations in the culture medium were measured by ELISA after the cells were cultured for 48 h. **(J)** ChIP analysis was performed with antibodies specific to H3K27me3 or the IgG control. Quantitative PCR was performed by using primers for the promoters of the *IL10* gene. The data shown are from three independent experiments. The data from B cells transfected with Jarid2-specific siRNA were compared with those from B cells transfected with control siRNA. *P < 0.05; **P < 0.01.

Furthermore, we wanted to explore the molecular mechanisms by which miR-155 regulates IL-10 expression in B cells. It has been reported that DNA-binding protein Jarid2 recruits polycomb repressive complex 2 (PRC2) to chromatin and inhibits gene expression in miR-155-deficient cells (28); however, this molecular mechanism has not been examined in B cells. Therefore, we speculated that miR-155 can activate IL-10 production by regulating Jarid2 and relieving the H3K27 histone methylation repression of IL-10. First, we sorted the B cell subsets according to their expression of CD24 and CD27 using FACS and then measured Jarid2 gene expression by Q-PCR; we found that CD24^hi^CD27^+^ B cells express much lower levels of Jarid2 than other B cell subsets (Figure 3A). We also analyzed Jarid2 expression in CD24^hi^CD27^+^ B cells in response to stimulation with CpG oligonucleotides over a time course and found that Jarid2 expression decreased with increasing stimulation time (Figure 3B). In addition, Jarid2 expression was negatively correlated with IL-10 gene expression in the CD24^hi^CD27^+^ B cells (Figure 3C). Furthermore, when the CD24^hi^CD27^+^ B cells were electroporated with miR-155 inhibitor, Jarid2 gene and protein levels were significantly increased compared to those in the control group (Figures 3D,E). These data indicate that miR-155 can modulate Jarid2 expression. We further sorted the IL-10^+^ and IL-10^−^ B cells using beads combined with FACS as previously described (29). We found that the IL-10^+^ B cells expressed much lower levels of Jarid2 than the IL-10^−^ B cells (Figure 3F). When Jarid2 expression was knocked down in the B cells and the cells were stimulated with CpG oligonucleotides for 48 h, the B cells produced much higher levels of IL-10 and TNFα than the control group (Figures 3G–I). It has been reported that Jarid2 can silence the transcription of its target genes through H3K27me3 (28). Therefore, we used ChIP-qPCR to determine whether Jarid2 directly targets H3K27me3 and regulates IL-10 production in B10 cells. As the data showed when the B cells were electroporated with Jarid2-specific siRNA and H3K27me3 was pulled down, the binding of H3K27me3 to the promoter of *IL10* was significantly reduced compared to that in the control group (Figure 3J). These data demonstrate that miR-155 activates IL-10 gene expression in B cells by regulating the Jarid2 target H3K27me3 and mediating repression.

### The Number of CD19^+^CD24^hi^CD27^+^ B Cells Was Decreased, and Their Function Was Impaired in CD Patients

**Figure 4:**
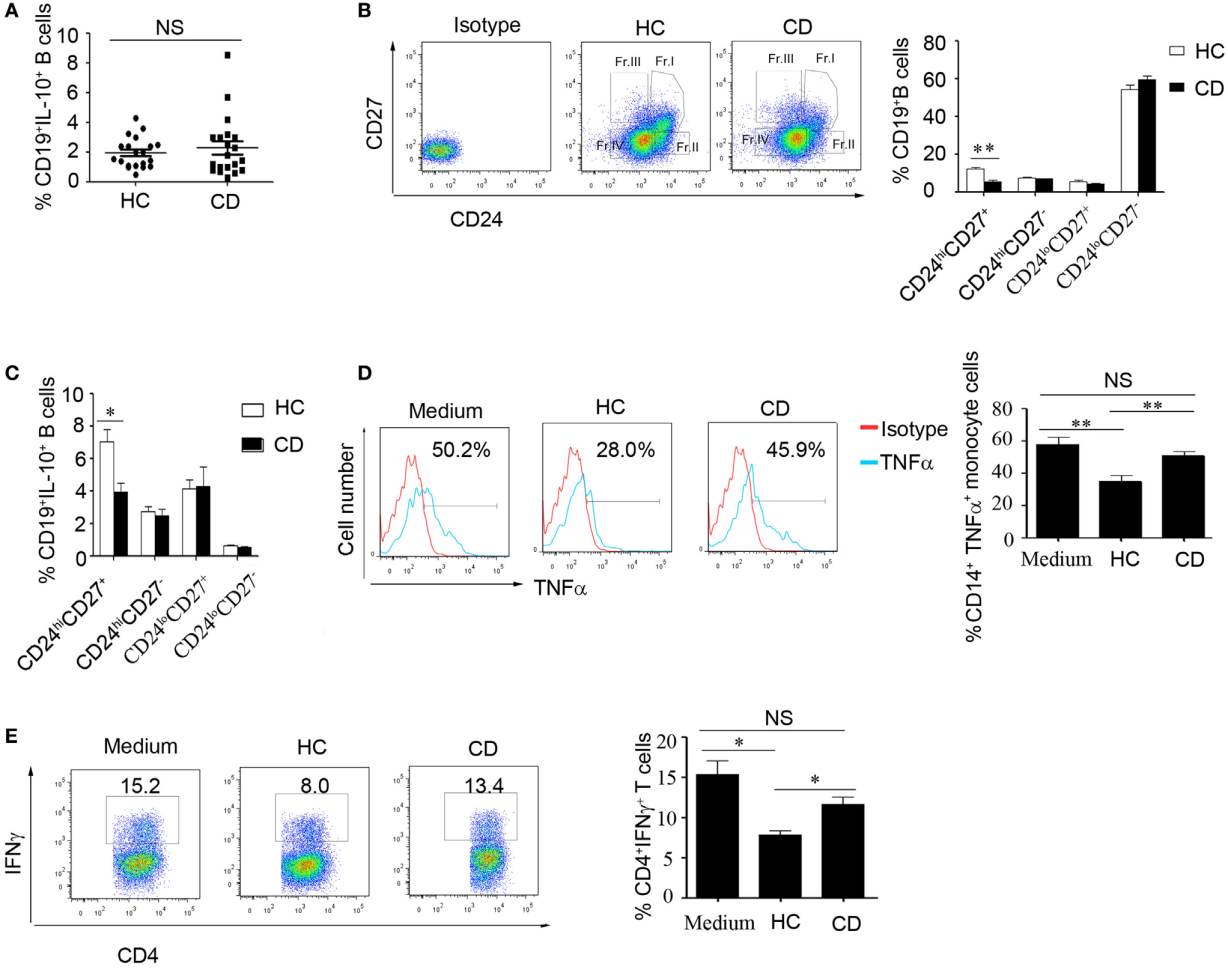
The number of CD19^+^CD24^hi^CD27^+^ B cells decreased, and their function was impaired in Crohn’s disease (CD) patients. **(A)** Freshly derived CD19^+^ B cells from CD patients and healthy controls (HCs) (*N* = 20 in both cases) were analyzed for interleukin-10 (IL-10) expression by FACS. The percentage of IL-10-secreting cells is shown (NS: no significant difference). **(B)** PBMCs from CD patients and HCs (*N* = 15) were analyzed for different B cell subsets using CD24 and CD27 markers, and the CD24 and CD27 antibodies isotype control are showed. The bars represent the mean ± SEM (right panel). **(C)** CD19^+^ B cells from HCs and CD patients (*N* = 15) were cultured with 100 nM CpG oligonucleotides for 48 h with the stimulation or inhibition cocktail added during the final 5 h of culture, and the percentages of IL-10^+^ B cells within the indicated B cell subsets were determined by FACS. The data are shown as the mean ± SEM. **(D)** Purified B cell subsets from the blood of HCs and CD patients were stimulated with 100 nM CpG oligonucleotides for 24 h and cultured with CD14^+^ monocytes isolated from HCs for 24 h; then, the cytoplasmic TNFα expression in CD14^+^ monocytes was assessed after 4 h of stimulation with 100 µg/mL LPS. The data are shown as the mean ± SEM (*N* = 5). **(E)** Purified B cell subsets from the blood of HCs and CD patients were stimulated with 100 nM CpG oligonucleotides for 24 h and cultured with CD3 mAb-stimulated CD4^+^CD25^−^ T cells isolated from HCs for 48 h. IFNγ expression in the CD4 T cells was analyzed by FACS. The data are shown as the mean ± SEM (*N* = 3). *P < 0.05; **P < 0.01; NS, no difference.

We then determined the number of B10 cells in CD patients. Interestingly, there was no significant difference in IL-10 production and Jarid2 mRNA expression in the B cells from the HC and CD groups (Figure 4A and Figure S3 in Supplementary Material). Next, we determined the percentages of B cell subsets using the markers CD24 and CD27 and found that the number of CD24^hi^CD27^+^ B cells were markedly reduced in the CD patients (12.43 ± 0.758 and 5.49 ± 0.912%, respectively) (Figure 4B). Additionally, the CD24^hi^CD27^+^ B cells from CD patients produce lower levels of IL-10 than those from the HCs (Figure 4C). Therefore, we isolated the CD24^hi^CD27^+^ B cells by FACS and conducted functional assays. We observed that when the CD24^hi^CD27^+^ B cells isolated from CD patients and HCs were stimulated with CpG oligonucleotides for 24 h, washed and cocultured with CD14^+^ monocytes, the CD24^hi^CD27^+^ B cells from the HCs could significantly inhibit TNFα production by the monocytes, while the CD24^hi^CD27^+^ B cells from the CD patients could not (Figure 4D). Additionally, when activated B cells were cocultured with purified CD4^+^ T cells, IFNγ production by the T cells was significantly reduced by the B cells from HCs but not by those from the CD patients (Figure 4E). Therefore, in the CD patients, not only was the number of CD24^hi^CD27^+^ B cells reduced, but their function was also impaired.

### Increased Frequency of TNFα-Producing CD19^+^CD24^hi^CD27^+^ B Cells in Patients with CD

**Figure 5:**
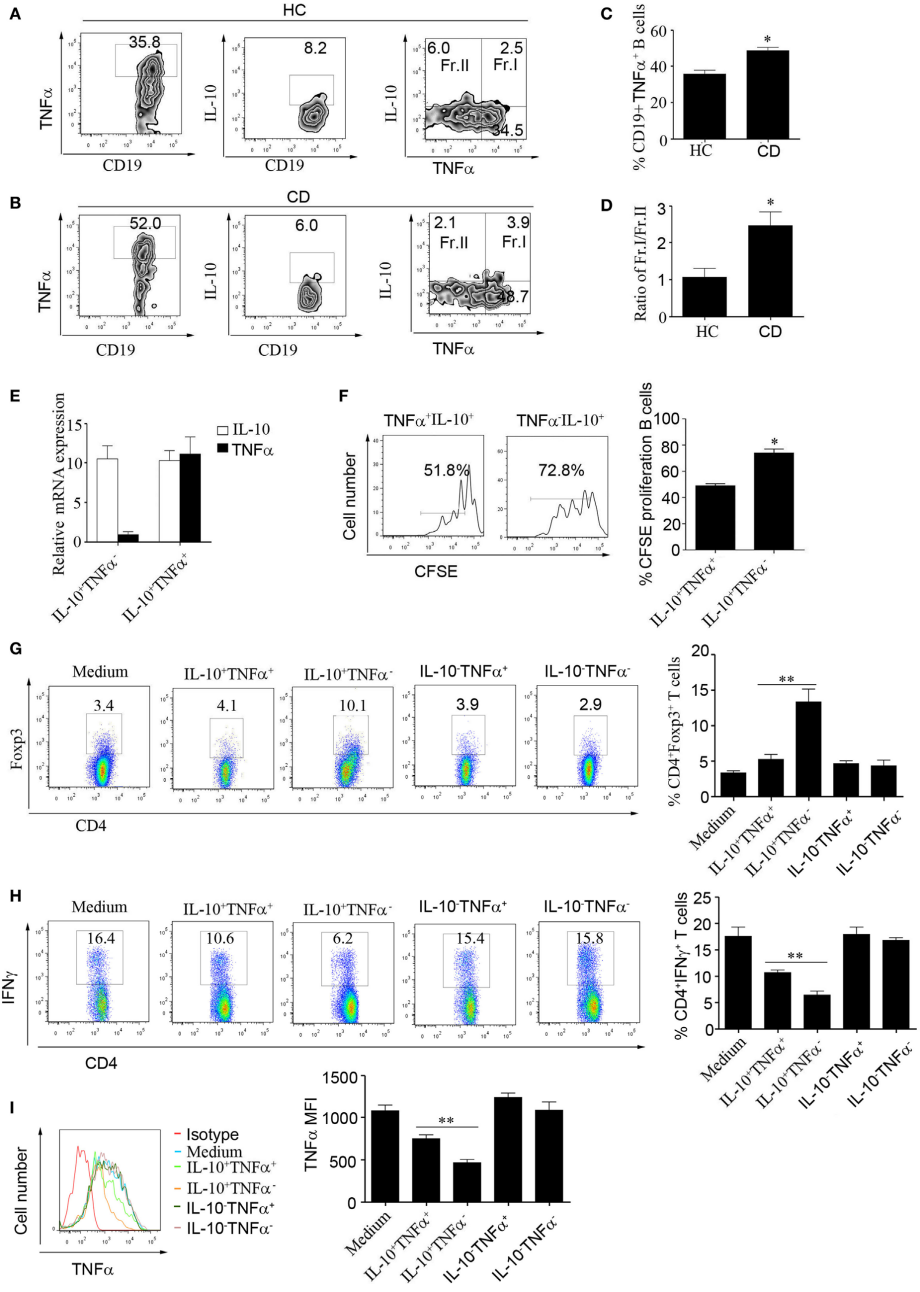
Increased frequencies of TNFα-producing CD19^+^CD24^hi^CD27^+^ B cells in patients with Crohn’s disease (CD). **(A–D)** CD19^+^CD24^hi^CD27^+^ B cells from healthy controls (HCs) and CD patients were sorted and stimulated with 100 nM CpG oligonucleotides for 48 h; then, FACS was used to measure TNFα and interleukin-10 (IL-10) expression in the cells from **(A)** HCs and **(B)** CD patients, *N* = 10. **(C)** The percentages of CD19^+^TNFα^+^ B cells from HCs and CD patients are shown. **(D)** The ratios of Fr.I (IL-10^+^TNFα^+^ B cells) to Fr.II (IL-10^+^TNFα^−^ B cells) from HCs and CD patients are shown. **(E)** Sorted CD19^+^CD24^hi^CD27^+^ B cells from HCs (*N* = 3) were stimulated with 100 nM CpG oligonucleotides for 48 h; then, the IL-10^+^TNFα^−^ B cells and IL-10^+^TNFα^+^ B cells were sorted, and the expression of IL-10 and TNFα was analyzed by Q-PCR. **(F)** Sorted IL-10^+^TNFα^−^ B cells and IL-10^+^TNFα^+^ B cells were labeled with CFSE and cultured with 100 nM CpG oligonucleotides for 72 h. The CFSE dilution was measured by FACS, and the percentages of CFSE-labeled proliferating B cells are shown (right panel). **(G–I)** FACS was used to sort IL-10^+^TNFα^-^, IL-10^+^TNFα^+^ IL-10^-^TNFα^+^ and IL-10^-^TNFα^−^ B cell subsets, which were then cultured with CD4^+^CD25^−^ T cells isolated from HCs and stimulated with 1 µg/mL anti-CD3 for 72 h. **(G)** Foxp3 expression and **(H)** IFNγ expression levels were measured by flow cytometry. In addition, CD14^+^ monocytes isolated from HCs were cultured for 24 h and then stimulated with 100 µg/mL LPS before the cytoplasmic TNFα expression was assessed. The data are shown as the mean ± SEM (*N* = 3). *P < 0.05, **P < 0.01.

We next wanted to explore the reason underlying the impaired function of CD24^hi^CD27^+^ B cells in CD patients. It has been reported that B cells can produce the inflammatory cytokine IL-6 and contribute to the pathogenesis of multiple sclerosis (30). In our study, we did not find an increase in IL-6 level in the B cells of CD patients (data not shown); however, interestingly, we found that CD24^hi^CD27^+^ B cells from CD patients could produce higher levels of TNFα than the cells from HCs. Additionally, the percentage of IL-10^+^/TNFα^+^ B cells was much higher, and the ratio of IL-10^+^/TNFα^+^ B cells to IL-10^+^/TNFα^−^ B cells was significantly increased in the CD patients (Figures 5A–D). We then sorted the IL-10^+^/TNFα^+^ and IL-10^+^/TNFα^−^ B cells using beads combined with FACS and determined their function. As shown by the data, the expression level of IL-10 mRNA was very high in both subsets, but that of TNFα mRNA was very low in the IL-10^+^/TNFα^−^ B cells (Figure 5E). Furthermore, we determined the proliferative ability of the two subsets and found that the IL-10^+^/TNFα^−^ B cells had a much higher proliferative ability than the IL-10^+^/TNFα^−^ B cells (Figure 5F). In addition, when the isolated subsets were cocultured with purified CD4^+^CD25^−^ T cells and stimulated with anti-CD3 for 3 days, the Foxp3 and IFNγ expression levels were analyzed, and the IL-10^+^/TNFα^−^ B cells demonstrated a greater ability to induce Foxp3 expression and a greater ability to inhibit IFNγ production by the CD4^+^CD25^−^ T cells (Figures 5G,H). Also, IL-10^+^/TNFα^−^ B cells could inhibit monocyte production of TNFα better than other B cell subsets (Figure 5I). These data suggest that higher numbers of IL-10^+/^TNFα^+^ B cells may contribute to the functional impairment of CD24^hi^CD27^+^ B cells in CD patients.

### miR-155 Induced CD19^+^CD24^hi^CD27^+^ B Cells to Produce Higher Levels of TNFα in CD Patients

**Figure 6:**
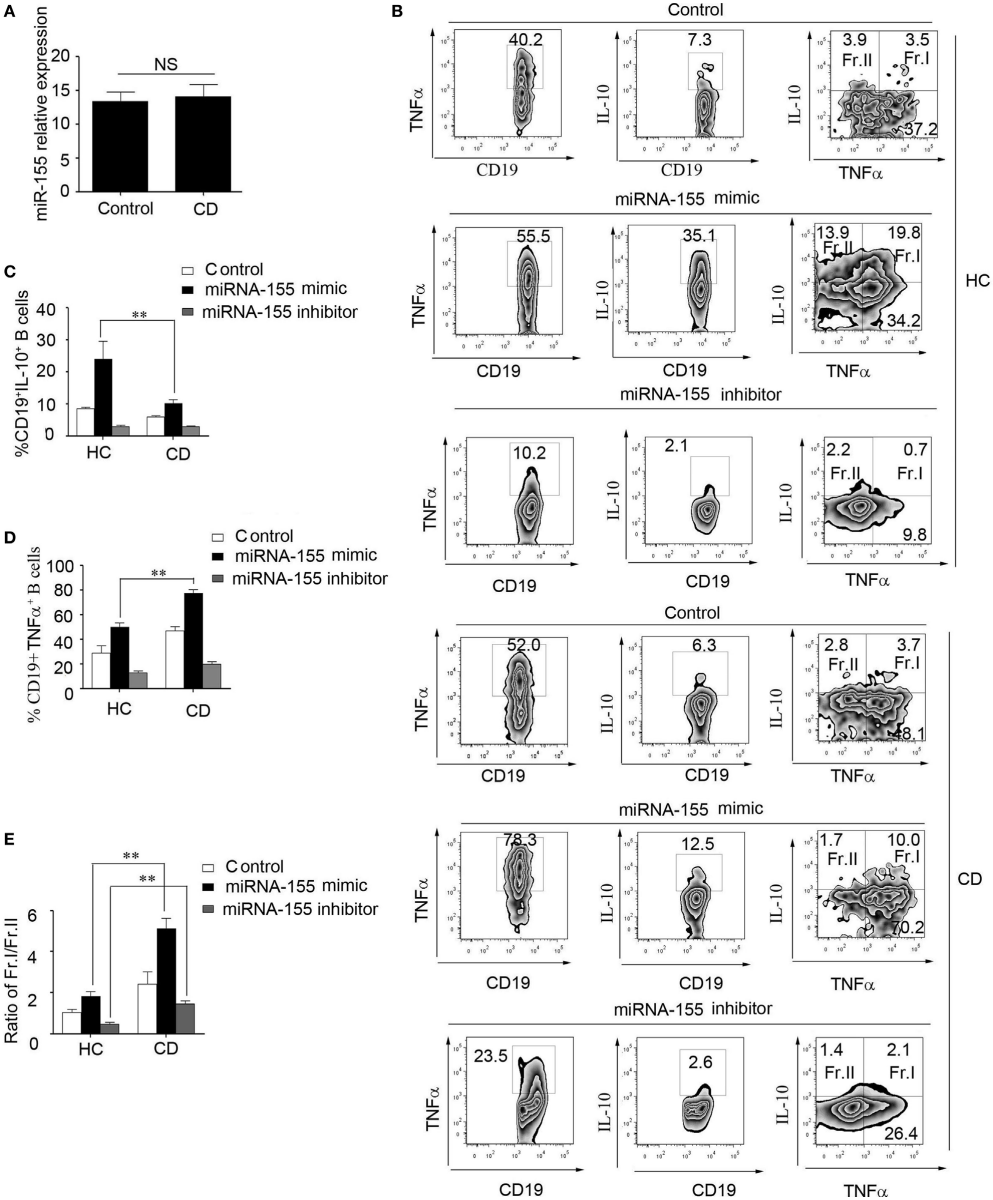
miR-155 induced CD19^+^CD24^hi^CD27^+^ B cells to produce higher levels of TNFα in Crohn’s disease (CD) patients. **(A)** CD19^+^CD24^hi^CD27^+^ B cells from healthy controls (HCs) and CD patients (*N* = 15) were sorted and stimulated with 100 nM CpG oligonucleotides for 24 h; then, miR-155 expression was determined by Q-PCR. **(B–E)** CD19^+^CD24^hi^CD27^+^ B cells from HCs and CD patients (*N* = 3) were sorted and electroporated with miR-155 mimic, inhibitor or control and cultured for another 48 h under stimulation with 100 nM CpG oligonucleotides. **(B)** Then, FACS was used to measure TNFα and interleukin-10 (IL-10) expression. The percentages of **(C)** CD19^+^IL-10^+^ B cells and **(D)** CD19^+^TNFα^+^ B cells from HCs and CD patients are shown. **(E)** In addition, the ratios of Fr.I (IL-10^+^TNFα^+^ B cells) to Fr.II (IL-10^+^TNFα^−^ B cells) from HCs and CD patients are shown.

To study the role of miR-155 in the function of CD19^+^CD24^hi^CD27^+^ B cells from CD patients, first, we determined the miR-155 expression in CD19^+^CD24^hi^CD27^+^ B cells. There was no significant difference between the HCs and CD patients (Figure 6A). We then transfected miR-155 mimic or inhibitor into the CD19^+^CD24^hi^CD27^+^ B cells from the HCs and CD patients, stimulated them with CpG oligonucleotides for 2 days and analyzed their IL-10 and TNFα production. The data showed that after the transduction of miR-155 mimic into the CD19^+^CD24^hi^CD27^+^ B cells, the production of both IL-10 and TNFα was increased in the HCs and CD patients, but the ratio of the IL-10^+^/TNFα^+^ B cells to IL-10^+^/TNFα^-^B cells in the CD patients was much higher than that in the control group (Figures 6B–E). When the CD19^+^CD24^hi^CD27^+^ B cells were transfected with the miR-155 inhibitor, the IL-10 and TNFα expression levels were decreased in both HC and CD groups. Interestingly, the ratio of IL-10^+^/TNFα^+^ B cells to IL-10^+^/TNFα^−^ B cells in the CD patients was still higher than that in the control group (Figures 6B–E). Therefore, in the CD patients, miR-155 could induce B cells to simultaneously secrete both IL-10 and a much higher level of TNFα, which may have reduced the function of CD19^+^CD24^hi^CD27^+^ B cells.

## Discussion

Abnormalities in IL-10-producing regulatory B cells contribute to the development and progression of autoimmune diseases (31). In humans, both CD19^+^CD24^hi^CD38^hi^ and CD19^+^CD24^hi^CD27^+^ regulatory B cells have been identified, but the regulation of IL-10 production by B cells is less clear. In our study, we found that the CD19^+^CD24^hi^CD27^+^ B cell subset highly expresses TLR9, which is more sensitive to stimulation by CpG oligonucleotides, and is the primary producer of IL-10 in healthy individuals and CD patients. In addition, Giltiay et al. recently reported that prestimulation of the B cells with TLR7 inducer IFNα was able to boost IL-10 production (32). In an attempt to further characterize the phenotype of CD19^+^CD24^hi^CD27^+^ B cells, we found that most CD24^hi^CD27^+^ B cells are CD21^+^ B cells, and they include very few of the unswitched memory (IgM^+^IgD^+^) B cells and cannot produce granzyme B (data not shown). Little is known about the potential link between miRNAs and B10 cells and their regulation in the pathology of CD. Our study demonstrated that the IL-10-producing CD24^hi^CD27^+^ B cells are regulated by miR-155 through the regulation of Jarid2-mediated repression regions in HCs. However, in patients with CD, in addition to the reduction in the number of CD24^hi^CD27^+^ B cells, the function of these B cells is impaired so that they cannot effectively inhibit innate and adaptive immunity. CD24^hi^CD27^+^ B cells in CD patients exhibit abnormally elevated secretion of TNFα and diminished secretion of IL-10, resulting in significantly increased frequencies of TNFα^+^/IL-10^+^ B cells compared to those in HCs. However, these cells neither efficiently induce Foxp3 expression nor inhibit IFNγ production in CD4^+^CD25^−^ T cells; TNFα^+^/IL-10^+^ B cells also had a decreased ability for inhibiting monocyte production of TNFα compared to TNFα^-^/IL-10^+^ B cells. miR-155 induced the B cells from CD patients to simultaneously produce IL-10 and high levels of TNFα, which may have been responsible for the reduced suppression function of CD24^hi^CD27^+^ B cells in CD patients. Interestingly, it has been reported that the pathogenic conversion of regulatory B10 cells into osteoclast-priming cells exacerbates osteoclast differentiation and bone destruction in rheumatoid arthritis (33). The cytokines produced by B cell subsets are associated with both pathogenic and protective roles in various immunological disorders. Therefore, in the inflammatory state, B10 cells may not always perform their protective functions.

B cells have great potential to regulate both innate and adaptive arms of the immune system by releasing cytokines. These cytokine-producing B cell subsets have multifunctional roles in health and diseases, playing pathologic as well as protective roles in autoimmunity, infection, and allergies (34). However, which B cells produce pro- and anti-inflammatory cytokines and the plasticity of B cell function depends on the environmental signals. B cell-derived cytokines, such as IL-6, IFNγ, and IL-17, influence the development of effector and memory CD4^+^ T cell responses (35). TNFα produced by B cells is necessary for the formation of lymphoid structures and the production of antigen-specific IgG1 (36). TNFα has been directly implicated in the pathogenesis of CD and plays have a crucial role in controlling intestinal inflammation and the associated clinical symptoms of CD (37, 38). Strategies for blocking TNFα are now commonly used as standard for CD in the clinic, and their mechanisms have been widely studied (39, 40). Interestingly, it remains unclear whether TNFα affects the number and function of regulatory B cells. In our study, we found that miR-155 could effectively induce CD24^hi^CD27^+^ B cells to produce IL-10 and the pro-inflammatory cytokine TNFα, which could have led to the loss of the suppressive and regulatory functions of B10 cells in CD patients. In addition, Timmermans et al. recently reported a reduction in IgM^+^ memory B cells in the blood of CD patients, which implies that the IgM response is impaired in the spleen and germinal center. However, transitional and CD21^low^ B cell numbers were increased in CD patients; therefore, the increase in transitional B cells could be compensating for the loss of mature B cells due to downregulation of CD21 (41). In addition, therapy with TNFα blockers restored the IgM memory B-cell generation and normalized transitional B-cell levels (41). Bankó et al. also reported that the increasing number of regulatory B10 cells might contribute to the therapeutic efficacy of anti-TNF agents in rheumatoid arthritis (42). Therefore, in future studies, we would like to determine whether the number and function of CD24^hi^CD27^+^ B cells can be recovered when CD patients are given infliximab therapy.

Many studies have focused on the role of miR-155 in the development of inflammatory diseases (43, 44), but the impact of miR-155 on B10 cells and CD has not been well studied. An important aspect of our study was to determine the effect that miR-155 exerts on the differentiation and function of B10 cells and further explored the molecular mechanism in HCs and CD patients. We found that miR-155 expression is correlated with IL-10 production in CD24^hi^CD27^+^ B cells. An increase in the expression of miR-155 induces IL-10 production, while a reduction in miR-155 expression inhibits IL-10 production in B cells. Our time course analysis showed that upon stimulation with CpG oligonucleotides, the expression of miR-155 and IL-10 increased, while that of Jarid2 decreased over time, with the highest expression levels of miR-155 and IL-10 occurring after 24 h, which supports the notion that miR-155 is critical for the production of IL-10 in CD24^hi^CD27^+^ B cells. It has been reported that TNFα production in response to anti-IgM antibody was decreased in miR-155-deficient mice (45). In addition, our data showed that miR-155 could induce TNFα production both in the HCs and CD patients, and inhibition of miR-155 could reduce both TNFα and IL-10 production; however, the ratio of TNFα^+^IL-10^+^/TNFα^−^IL-10^+^ B cells was still much higher in the CD patients. These data suggest that miR-155 may cause a differential effect on cytokine production that induces the higher TNFα production that contributes to disease development in CD patients.

Taken together, our study reveals a novel molecular mechanism for the regulation of IL-10-producing B cells that is mediated by the direct inhibition of Jarid2 by miR-155 and the associated decrease in H3K27me3 binding to the *IL10* promoter to promote IL-10 expression. Additionally, we demonstrated that the function of CD24^hi^CD27^+^ B cells was impaired in CD patients, likely due to the miR-155-induced production of TNFα by CD24^hi^CD27^+^ B cells, resulting in an increase in the frequency of IL-10^+^TNFα^+^ B cells, which contribute to the functional impairment of CD24^hi^CD27^+^ B cells. These results provide insight into the mechanisms by which miR-155 regulates IL-10 production in CD24^hi^CD27^+^ B cells and encourage the development of B10 cell-based strategies to relieve the symptoms of Crohn’s disease.

## Ethics Statement

All the patients provided written informed consent to participate in the study, which was carried out in accordance with the principles of the Declaration of Helsinki and was approved by the ethics committee of Xinhua Hospital.

## Author Contributions

YZ and LS designed and discussed the study. YZ carried out most of the experiments and collected and analyzed the data. WG and MY recruited study participants and provided clinical samples. WW helped with cell sorting. YZ, LH, YM, GX, BB, LL, and LS contributed to writing the article.

## Conflict of Interest Statement

The authors declare that the research was conducted in the absence of any commercial or financial relationships that could be construed as a potential conflict of interest.
