# Supplementary material for: miR-155 Regulates IL-10-Producing CD24hiCD27+ B Cells and Impairs Their Function in Patients with Crohn’s Disease
Source: Front Immunol. 2017 Aug 3;8:914. doi: 10.3389/fimmu.2017.00914 (PMC5540954; doi:10.3389/fimmu.2017.00914)
Supplement: Supplementary file 1 [file data_sheet_1.pdf]

MiR-155 regulates IL-10-producing $CD24^{hi}CD27^{+}$ B cells and impairs their function in patients with Crohn's disease

Yingxia Zheng, [1, 2] Wensong Ge, [3] Yanhui Ma, [1] Guohua Xie, [1] Weiwei Wang, [1] Li Han, [1] Bingxian Bian, [1] Li Li, [1] Lisong Shen [1,]*

Supplementary information

miRNA Microarray

Human $CD19^{+}$ B cells were isolated and were stimulated with CpG oligonucleotides for 48 h. Total RNA was quantified by the NanoDrop ND-2000 (Thermo Scientific) and the RNA integrity was assessed using Agilent Bioanalyzer 2100 (Agilent Technologies).The sample labeling, microarray hybridization and washing were performed based on the manufacturer's standard protocols. Briefly, total RNA were dephosphorylated, denaturated and then labeled with Cyanine-3-CTP. After purification the labeled RNAs were hybridized onto the microarray. After washing, the arrays were scanned with the Agilent Scanner G2505C (Agilent Technologies). Feature Extraction software (version10.7.1.1, Agilent Technologies) was used to analyze array images to get data.

Cell viability

Isolated B cells were electroporated with control or miR-155 mimic and miR-155 stimulated with 100 nM CPG and cultured for another 48 h. The viability of the cells was analyzed by CellTiter-Glo luminescent assay (Promega, Madison, WI); the luminescence values were measured with microplate computer software (Bio-Rad Laboratories).

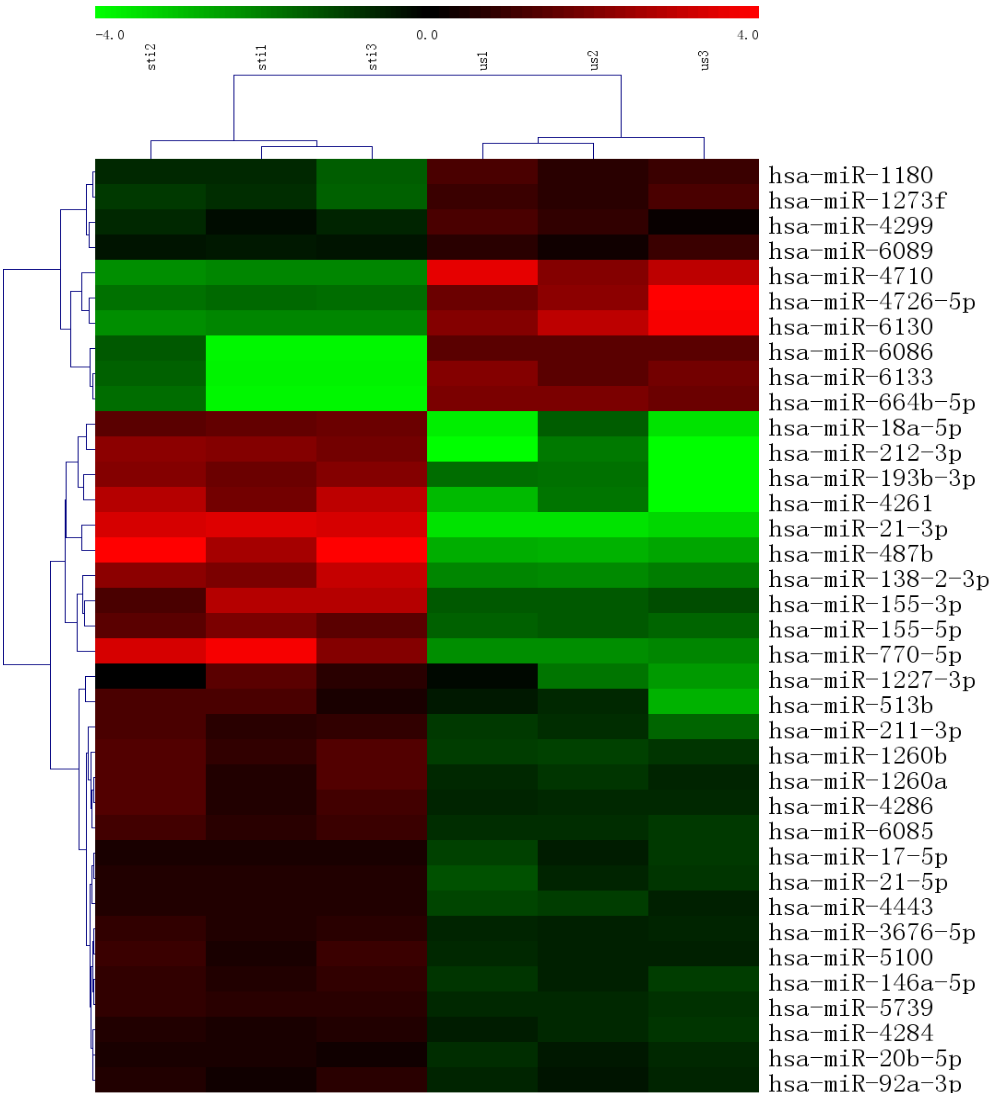

Supplementary figure 1: Heat map and unsupervised hierarchical clustering of miRNAs in human $CD19^+$ B cells stimulated with or without 100nM CpG oligonucleotides for 48 h. The microRNA arrays were scanned with the Agilent Scanner G2505C. Differentially expressed miRNAs were then identified through fold change. Hierarchical Clustering was performed to show the distinguishable miRNAs expression pattern among samples.

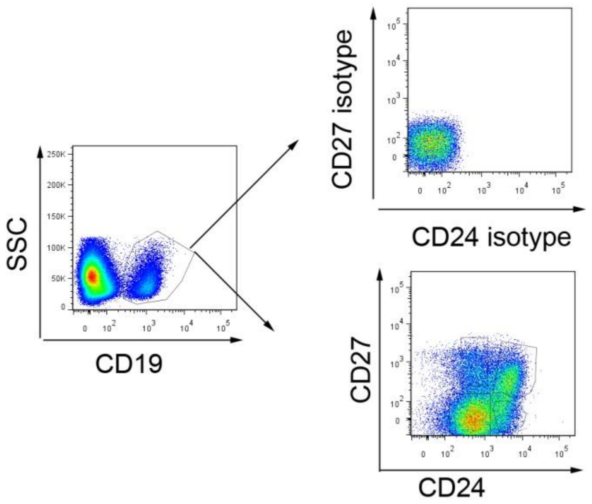

Supplementary figure 2: Flowcytometric gating strategy for B cell subsets in a representative healthy control. B cells defined as $CD19^{+}$ lymphocytes were further classified into four subsets ($CD24^{hi}CD27^{+}$; $CD24^{lo}CD27^{+}$; $CD24^{hi}CD27^{-}$; $CD24^{lo}CD27^{-}$) according to the CD24 and CD27 markers.

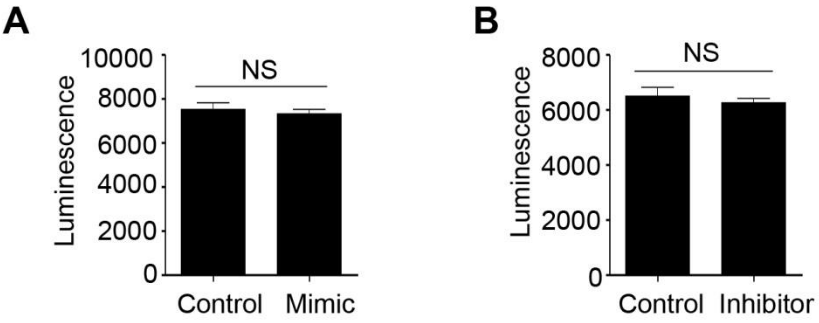

Supplementary Figure 3: (A) Isolated $CD19^{+}$ B cells were electroporated with control or miR-155 mimic; then, the cells were cultured in a 96-well microplate (2 x 10^{5} cells per well), stimulated with 100 nM CPG and cultured for another 48 h. The viability of the cells was then analyzed. (B) FACS was used to sort $CD24^{hi}CD27^{+}$ B cells, which were electroporated with control or miR-155 inhibitor; then, the cells were cultured in a 96-well microplate (2 x 10^{5} cells per well), stimulated with 100 nM CPG and cultured for another 48 h. The viability of the cells was then analyzed. NS: no significant difference.

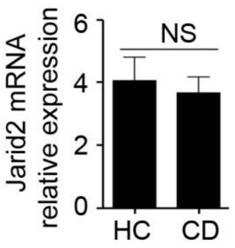

Supplementary Figure 4: CD19$^+$B cells isolated from HCs and CD patients; Jarid2 expression levels were measured by Q-PCR. NS: no significant difference.
